# Multiple modes of antigen exposure induce clonotypically diverse epitope-specific CD8^+^ T cells across multiple tissues in nonhuman primates

**DOI:** 10.1371/journal.ppat.1010611

**Published:** 2022-07-07

**Authors:** Jennifer Simpson, Carly E. Starke, Alexandra M. Ortiz, Amy Ransier, Sam Darko, Daniel C. Douek, Jason M. Brenchley

**Affiliations:** 1 Barrier Immunity Section, Lab of Viral Diseases, Division of Intramural Research, NIAID, NIH, Bethesda, Maryland, United States of America; 2 Human Immunology Section, Vaccine Research Center, NIAID, NIH, Bethesda, Maryland, United States of America; Oregon National Primate Research Center, UNITED STATES

## Abstract

Antigen-specific CD8^+^ T cells play a key role in the host’s antiviral response. T cells recognize viral epitopes via the T cell receptor (TCR), which contains the complementarity-determining region-3 (CDR3), comprising the variable, diversity and joining regions of the TCRβ gene. During chronic simian immunodeficiency virus (SIV) infection of Asian macaque nonhuman primates, tissue-specific clonotypes are identifiable among SIV-specific CD8^+^ T cells. Here, we sought to determine level of antigen exposure responsible for the tissue-specific clonotypic structure. We examined whether the priming event and/or chronic antigen exposure is response for tissue-specific TCR repertoires. We evaluated the TCR repertoire of SIV-specific CD8^+^ T cells after acute antigen exposure following inoculation with a SIV DNA vaccine, longitudinally during the acute and chronic phases of SIV, and after administration of antiretrovirals (ARVs). Finally, we assessed the TCR repertoire of cytomegalovirus (CMV)-specific CD8^+^ T cells to establish if TCR tissue-specificity is shared among viruses that chronically replicate. TCR sequences unique to anatomical sites were identified after acute antigen exposure via vaccination and upon acute SIV infection. Tissue-specific clones also persisted into chronic infection and the clonotypic structure continued to evolve after ARV administration. Finally, tissue-specific clones were also observed in CMV-specific CD8^+^ T cells. Together, these data suggest that acute antigen priming is sufficient to induce tissue-specific clones and that this clonal hierarchy can persist when antigen loads are naturally or therapeutically reduced, providing mechanistic insight into tissue-residency.

## Author summary

During viral infection, CD8^+^ T cells that bind a specific viral particle through their T cell receptor (TCR) can help control viral replication. Infection with simian immunodeficiency virus (SIV) in nonhuman primates is a commonly used animal model of HIV infection. Here we assess the TCR sequences of CD8^+^ T cells specific for the SIV gag gene during vaccination with an experimental SIV vaccine and throughout SIV infection, including during treatment with antiretroviral drugs. We identified unique TCR sequences in specific tissues, which were not identified in the blood or in other tissues, both in response to vaccination and throughout SIV infection with and without antiretroviral treatment. We also observed tissue-specific TCR sequences in CD8^+^ T cells specific for Cytomegalovius, another virus that causes a chronic infection in humans. Together, our findings identify the conditions required to form a tissue-specific TCR repertoire.

## Introduction

Virus-specific CD8^+^ T cells are critical for control of SIV and HIV viral replication [1–3]. Upon HIV infection, antigen-specific CD8^+^ T cells mobilize in the blood by approximately 20 days after infection [4]. Similarly, SIV infection in nonhuman primates induces antigen-specific CD8^+^ T cells in the blood and gut by approximately 14 days post infection [5]. While SIV-specific CD8^+^ T cells contribute to reduction of viral replication throughout viral infection [3,6], virus-specific CD8^+^ T cells seem to preferentially exhibit cytolytic capabilities in early acute infection [7] and non-cytolytic function during chronic infection [3].

SIV-specific CD8^+^ T cells recognize viral antigens via a hypervariable complementarity-determining region-3 (CDR3) on the T cell receptor (TCR). Together, the CDR3 sequences of every T cell comprises the TCR repertoire. Utilizing a rhesus macaque (*Macaca mulatta*) SIV-infection model, it was determined that a diverse TCR repertoire among SIV-specific CD8^+^ T cells is associated with reduced viral escape, whereas more conserved TCR repertoires are associated with impaired restriction of viral replication, with viral escape mutations more frequently emerging [8]. Some TCR clonotypes of CD8^+^ T cells with the same epitope specificity can be shared by multiple individuals—termed public clonotypes—and their frequency tends to be associated with better control of SIV replication *in vivo* [8]. More recently, we have shown that within an individual, public clonotypes among SIV-specific CD8^+^ T cells are more prone to be present in multiple anatomical sites compared to private clonotypes [9].

However, the degree to which tissue-specific clonotypic hierarchies is specific to SIV is unclear. Cytomegalovirus (CMV) is a common herpesvirus, where approximately 45–100% of the world’s adult population exhibits CMV seropositivity [10]. Similar to other herpesviruses, CMV establishes a chronic infection in the host, leading to periods of latent infection and reactivation [11]. CMV-specific CD8^+^ T cells rapidly increase upon acute CMV infection and have been shown to increase over time. CD8^+^ T cells specific for CMV immunodominant epitopes, such as immediate early 1 (IE1), exhibit cytotoxic and polyfunctional phenotypes and positively correlate with serum CMV-specific IgG levels [12]. Analyses of the TCR repertoire of CMV-specific CD8^+^ T cells present in peripheral blood have been conducted in humans and animal models, but most analyses are conducted *ex vivo* under peptide stimulation [13, 14]. Therefore, the tissue specificity of the CMV-specific CD8^+^ T cell repertoire is unknown in natural CMV infection.

Active SIV and CMV infections exhibit chronic antigenic stimulation *in vivo*, in contrast to responses against vaccination or to viruses which only acutely replicate. Several experimental vaccines have been shown to induce SIV-specific CD8^+^ T cells at mucosal sites in addition to the periphery [15–17], but whether the TCR repertoire of these antigen-specific CD8^+^ T cells is similar or distinct to those induced by chronic antigen stimulation is unknown.

Here, we sought to determine the kinetics underlying the tissue-specific distribution of TCR repertoires of antigen-specific CD8^+^ T cells. Using multiple experimental models, we establish that both the initial antigen priming event and chronic antigen exposure are individually sufficient to induce tissue-specific TCR signatures and that clonotypes can continue to evolve in SIV-infected animals treated with antiretrovirals (ARVs). These data provide mechanistic insight into tissue-residency.

## Results

### SIV-specific CD8^+^ T cells exhibit tissue-specific TCR repertoires upon acute infection, which evolve throughout chronic infection

SIV-infected rhesus macaques expressing the MHC-I allele Mamu A*01 commonly present the immunodominant SIV-gag epitope CM9 (CTPYDINQM) [18,19]. To characterize the CM9-specific CD8^+^ T cell response, we utilized a fluorochrome-conjugated MHC-I pentamer containing the CM9 sequence to identify and sort CM9-specific CD8^+^ T cells. In animals intrarectally challenged with SIVmac239X or infected i.v with SIVmac239, CM9-specific CD8^+^ T cell frequencies were similar from acute to chronic infection and did not dramatically decrease after treatment with ARVs in the peripheral blood mononuclear cells (PBMCs), lymph nodes (LNs) or bronchoalveolar lavage (BAL) (S1A Fig).

**Fig 1:**
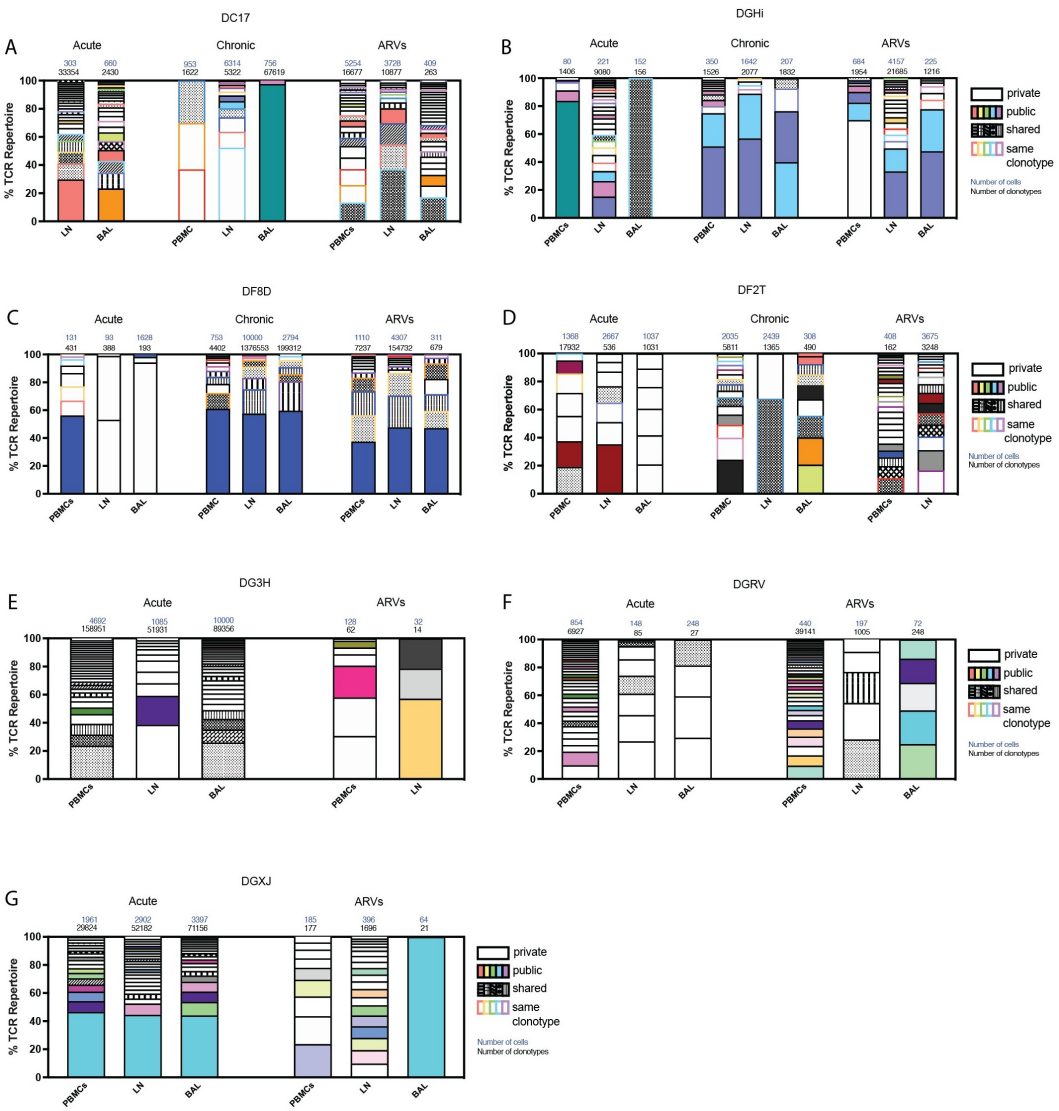
SIV-specific CD8^+^ T cell repertoire fluctuates throughout infection and during ARV treatment. PBMCs, LN biopsies and BAL were sampled from 7 different SIVmac239X or SIVmac239-infected rhesus macaques during acute infection, chronic infection and after 2–7 months of ARV treatment. (A-G). Clonotypes consisting of more than 1% of the TCR repertoire were represented as percentage of the TCR repertoire during acute and chronic infection and with ARV treatment. “Public” clonotypes are the same clonotype (same V and J segments and same CDR3 amino acid sequence) found in multiple animals in this study, and matching clonotypes previously identified in the VDJdb database. “Shared” clonotypes were those found only in one animal but observed in multiple tissues. “Private” clonotypes were identified in a single anatomical site in a single animal. n = 7 animals. Total cell and TCR sequences numbers are listed above each column.

To establish how or if the clonotypic hierarchy of CM9-specific CD8^+^ T cells evolves throughout infection, we assessed the TCRβ sequences in animals longitudinally sampled through acute and chronic infection and after several months of ARV treatment. During acute infection, public clonotypes (shared between multiple animals) and shared clones (shared between multiple anatomical sites within the same animal at the same time point) were identified in four animals (Fig 1A–1G). Clonotypes unique to one tissue (private clonotypes) were also present in acute infection, suggesting antigen priming was sufficient for induction of tissue-specific CM9-specific CD8^+^ T cells. TCRβ sequences assessed after the animals transitioned into chronic infection similarly included private, shared, and public clones, as did those from animals treated with ARVs, indicating that reduced antigen presence associated with ARV treatment does not substantially alter the tissue-specificity of CM9-specific CD8^+^ T cells. These data suggest that clonotypes in a single anatomical site can be initiated early after antigenic-exposure, can persist over time, and can continue to evolve as antigenic-exposure continues.

**Fig 2:**
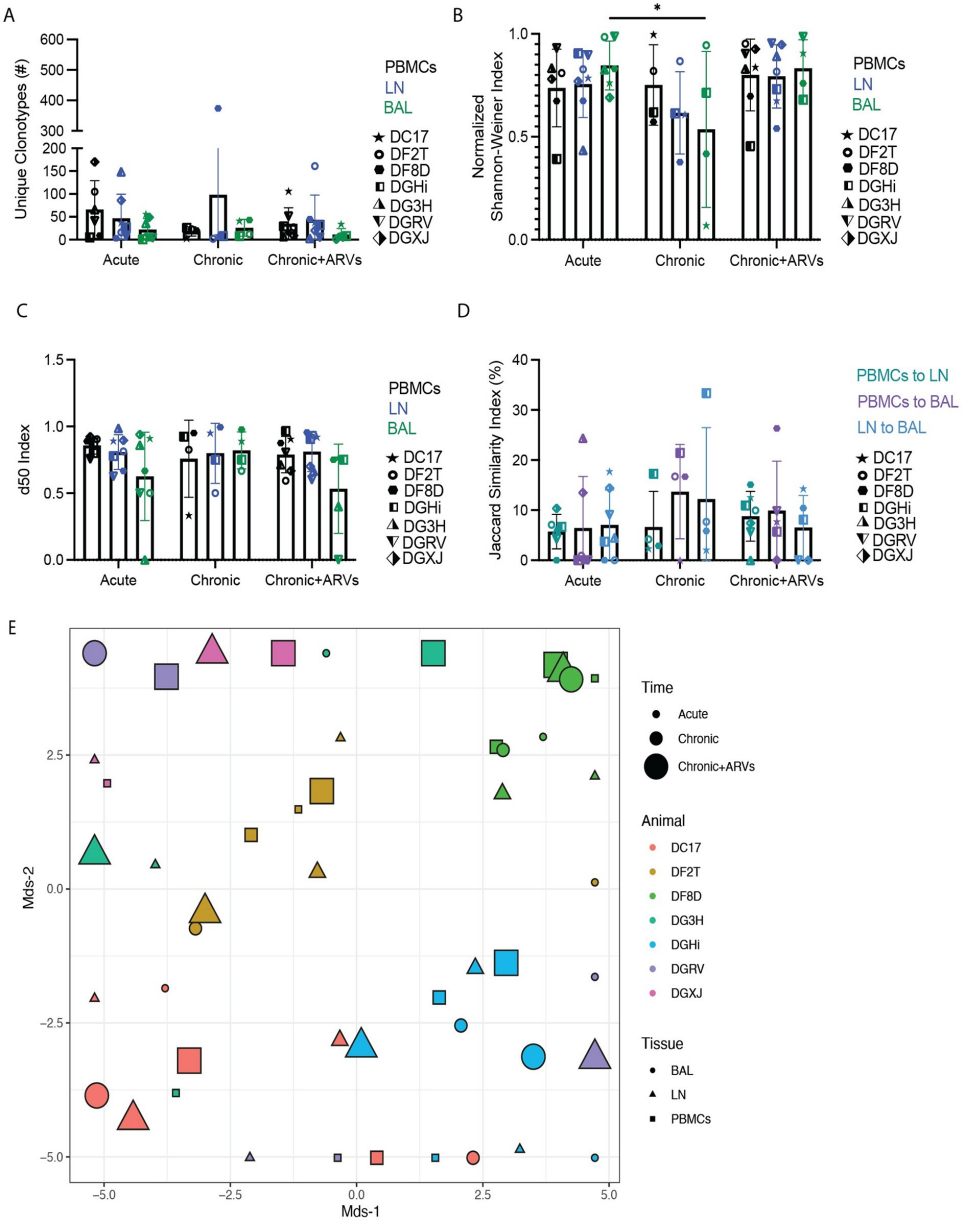
SIV-specific CD8^+^ T cells exhibit similar TCR diversity and similarity throughout SIV infection. PBMCs, LN biopsies and BAL were sampled from SIVmac239X or SIVmac239-infected rhesus macaques during acute infection, chronic infection and after 2–6 months of ARV treatment. (A) The number of unique clonotypes in multiple anatomical sites at all timepoints. (B) The normalized Shannon-Weiner diversity index for the TCR repertoires of all tissues throughout SIV infection. (C) The d50 diversity index for the TCR repertoires of all tissues throughout SIV infection. (D) The Jaccard similarity index for comparisons between each tissue’s TCR repertoire. In (A-D), data is represented by mean and SD, with each individual animal represented by a unique symbol. (E) MDS plot of the TCR repertoires of each animal, tissue and timepoint throughout infection. Two-way ANOVA was used to determine statistical significance for panels A to D. n = 7 animals.

The diversity of the TCR can fluctuate throughout infection and ARV treatment can induce a narrowing of the TCR repertoire [20]. Comparing the number of unique clonotypes and using two measures of diversity, the Shannon-Weiner index and the d50 index, we did not observe any significant differences in TCR repertoire diversity of CM9-specific CD8^+^ T cells, with the exception of a significantly higher normalized Shannon-Weiner index in BAL at the acute compared to the chronic timepoint (Fig 2A–2C). Singular V and J segments from CM9-specific CD8^+^ T cells across all time points were plotted in heat maps and showed substantial diversity at each time point, with minimal clustering by time point (S1B and S1C Fig). We utilized the Jaccard similarity index to identify how similar or distinct the clonotypic repertoires of different anatomical sites are to each other. We observed no significant differences between the Jaccard similarity indexes for the comparisons at any time point (Fig 2D), suggesting that unique and shared clonotypes are prevalent in equal amounts throughout the course of infection and after ARV treatment. Multidimensional scaling (MDS) plots of all samples were used to evaluate if any clustering was present regarding the different time points and anatomical sites. While there was minimal clustering by animal, no clear cluster patterns were observed regarding either anatomical site or time point (Fig 2E).

### SIV-gag DNA vaccine induces tissue-specific CM9-specific CD8^+^ T cells with public clonotypes

**Fig 3:**
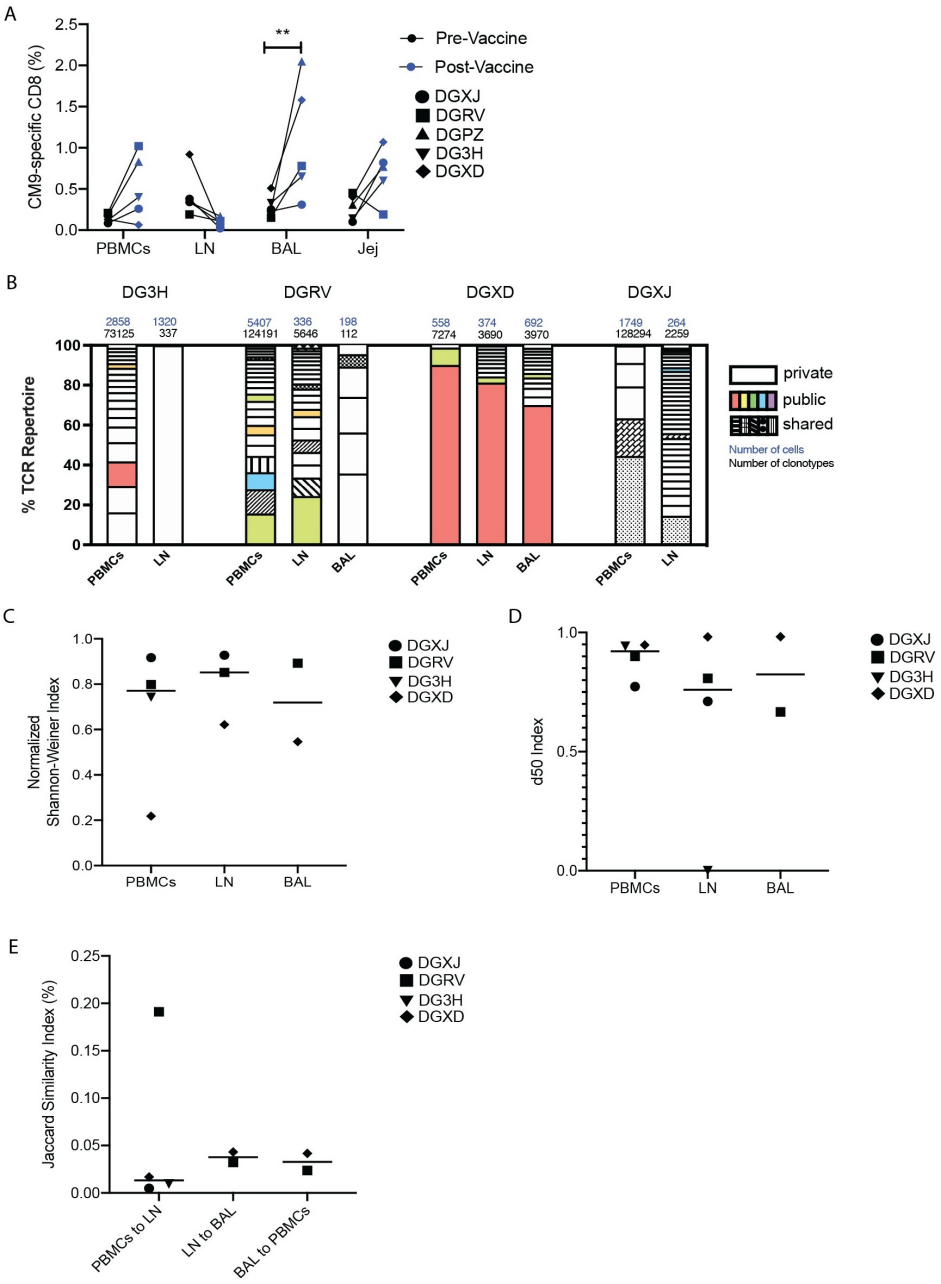
SIV-gag DNA vaccine induced a diverse TCR repertoire and tissue-specific clonotypes. PBMCs, BAL, LN, and jejunum (jej) biopsies were sampled from rhesus macaques who had been administered with SIV-gag DNA vaccine. (A) CM9-specific CD8^+^ T cells in all tissues pre- and post- vaccine administration as a percentage of total CD8^+^ cells. (B) Clonotypes consisting of more than 1% of the TCR repertoire are represented as percentage of the TCR repertoire. “Public” clonotypes are the same clonotype (same V and J segments and same CDR3 amino acid sequence) found in multiple animals in this study, and matching clonotypes previously identified in the VDJdb database. “Shared” clonotypes were those found only in one animal but observed in multiple tissues. “Private” clonotypes were identified in a single anatomical site in a single animal. Total cell and TCR sequences numbers are listed above each column. (C) The normalized Shannon-Weiner diversity index for the TCR repertoires of all tissues. (D) The d50 diversity index for the TCR repertoires of all tissues. (E) The Jaccard similarity index for comparisons between each tissue’s TCR repertoire. In (C) to (E), data are presented as mean, with individual data points shown. Two-way ANOVA (A) and one-way ANOVA (C-E) were used to determine statistical significance. n = 5 animals.

As tissue-specific clonotypes were induced upon acute infection, we sought to determine if controlled and limited antigen exposure, induced by a SIV-gag vaccine, would be sufficient to induce a similar clonal hierarchy of CM9-specific CD8^+^ T cells. Rhesus macaques were administered five doses of 1mg plasmid DNA construct containing the SIV-gag gene, which has been previously shown to induce CM9-specific CD8^+^ T cells in peripheral blood and peripheral LNs of rhesus macaques [16]. Ten days after the final vaccine dose, CM9-specific CD8^+^ T cell frequencies were significantly higher in the BAL and not in other anatomical sites (Fig 3A), suggesting the SIV-gag DNA vaccine regimen was insufficient to induce long-lasting CM9-specific CD8^+^ T cells across multiple tissues.

Assessment of the TCR repertoire of vaccine-induced CM9-specific CD8^+^ T cells from multiple anatomical sites revealed public and shared clonotypes, in addition to clonotypes unique to a single tissue (Fig 3B). These data demonstrate that the same clonotypic hierarchies we observe across multiple anatomical sites after SIV infection were present with the use of the DNA vaccine. Diversity and similarity indexes revealed similar diversity (Fig 3C and 3D) and tissue-specificity (Fig 3E) for each anatomical site. While individual V and J segment usage was not associated with any particular anatomical site (S2A and S2B Fig), some minor clustering by individual animal was observed by MDS analysis (S2C Fig). Together, these data imply that limited antigenic exposure is sufficient to induce similarly diverse and unique TCR repertoires in multiple anatomical sites, similar to what was observed upon SIV infection.

### Chronic antigen exposure induces tissue-specific clonotypes in a virus-independent manner

**Fig 4:**
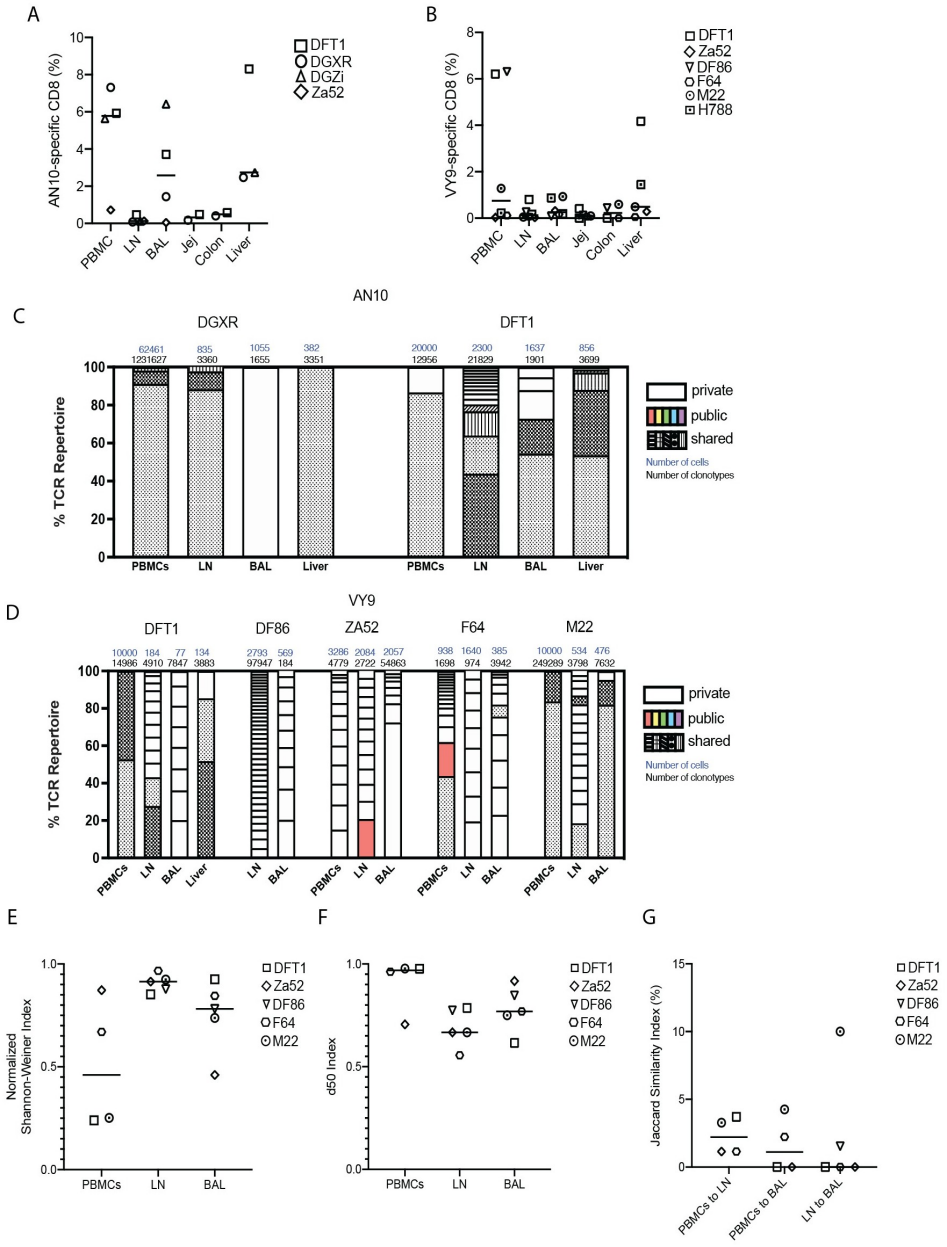
CMV-specific CD8^+^ T cells exhibit public, shared and tissue-specific clonotypes upon natural infection. PBMCs, LN, liver biopsies, and BAL were sampled from rhesus macaques who had been naturally infected with CMV. (A) The number of AN10-specific CD8^+^ T cells in multiple anatomical sites as a percentage of total CD8^+^ cells. (B) The number of VY9-specific CD8^+^ T cells in multiple anatomical sites as a percentage of total CD8^+^ cells. (C) Clonotypes consisting of more than 1% of the TCR repertoire of AN10-specific CD8^+^ T cells are represented as a percentage of the total TCR repertoire. “Public” clonotypes are the same clonotype (same V and J segments and same CDR3 amino acid sequence) found in multiple animals in this study, and matching clonotypes previously identified in the VDJdb database. “Shared” clonotypes were those found only in one animal but observed in multiple tissues. “Private” clonotypes were identified in a single anatomical site in a single animal. Total cell and TCR sequences numbers are listed above each column. (D) Clonotypes consisting of more than 1% of the TCR repertoire of VY9-specific CD8^+^ T cells are represented as a percentage of the total TCR repertoire. (E) The normalized Shannon-Weiner diversity index for the TCR repertoires of VY9-specific CD8^+^ T cells. (F) The d50 diversity index for the TCR repertoires of VY9-specific CD8^+^ T cells. (G) The Jaccard similarity index for comparisons between each tissue’s VY9-specific CD8^+^ T cell repertoire. In (A), (B) and (E)-(G), data are presented as mean, with individual data points. One-way ANOVA or mixed effects analysis was used to determine statistical significance. n = 2 to 5 animals.

To determine whether the existence of tissue-residency among virus-specific CD8^+^ T cells was unique to SIV, we conducted similar studies with cytomegalovirus (CMV)-infected animals and analyzed CMV-specific CD8^+^ T cell clonal hierarchy across multiple tissues. All the anatomical sites previously assessed in SIV infected animals were sampled, as was the liver, as CMV specific CD8^+^ T cells are commonly found in the liver [21]. CMV induces a chronic infection in the host and can lead to disease in immunocompromised individuals [22,23]. To assess the TCR repertoire of CMV-specific CD8^+^ T cells, we sampled rhesus macaques expressing the Mamu A*02 allele and who had acquired CMV infection naturally. CMV-specific CD8^+^ T cells were identified by MHC-I tetramers containing the Mamu A*02 restricted immunodominant epitopes AN10 (TTRSLEYKN) and VY9 (VTTLGMALY) [24]. There were no statistically significant differences in the frequencies of AN10- or VY9-specific CD8^+^ T cells between different anatomical sites (Fig 4A and 4B) in the animals we studied, though they tended to be lower in LNs compared to other anatomical sites. The TCR repertoire of AN10-specific CD8^+^ T cells featured clonotypes that were shared between multiple tissues and a small number of tissue-specific clonotypes (Fig 4C). VY9-specific CD8^+^ T cells had multiple tissue-specific clonotypes and both shared and public clonotypes (Fig 4D). Further analysis of the TCR repertoire of VY9-specific CD8^+^ T cells showed no clear groupings of specific V and J segment usage by tissue (S3A and S3B Fig). Similarly, VY9-specific CD8^+^ TCR repertoire showed similar diversity between different tissues as determined by Shannon diversity and d50 indexes (Fig 4E and 4F). The low Jaccard similarity indexes indicate similarly unique repertoires between different anatomical sites (Fig 4G).

### Similar clonotypic diversity and similarity among tissues between SIV-specific and CMV-specific CD8^+^ T cells

**Fig 5:**
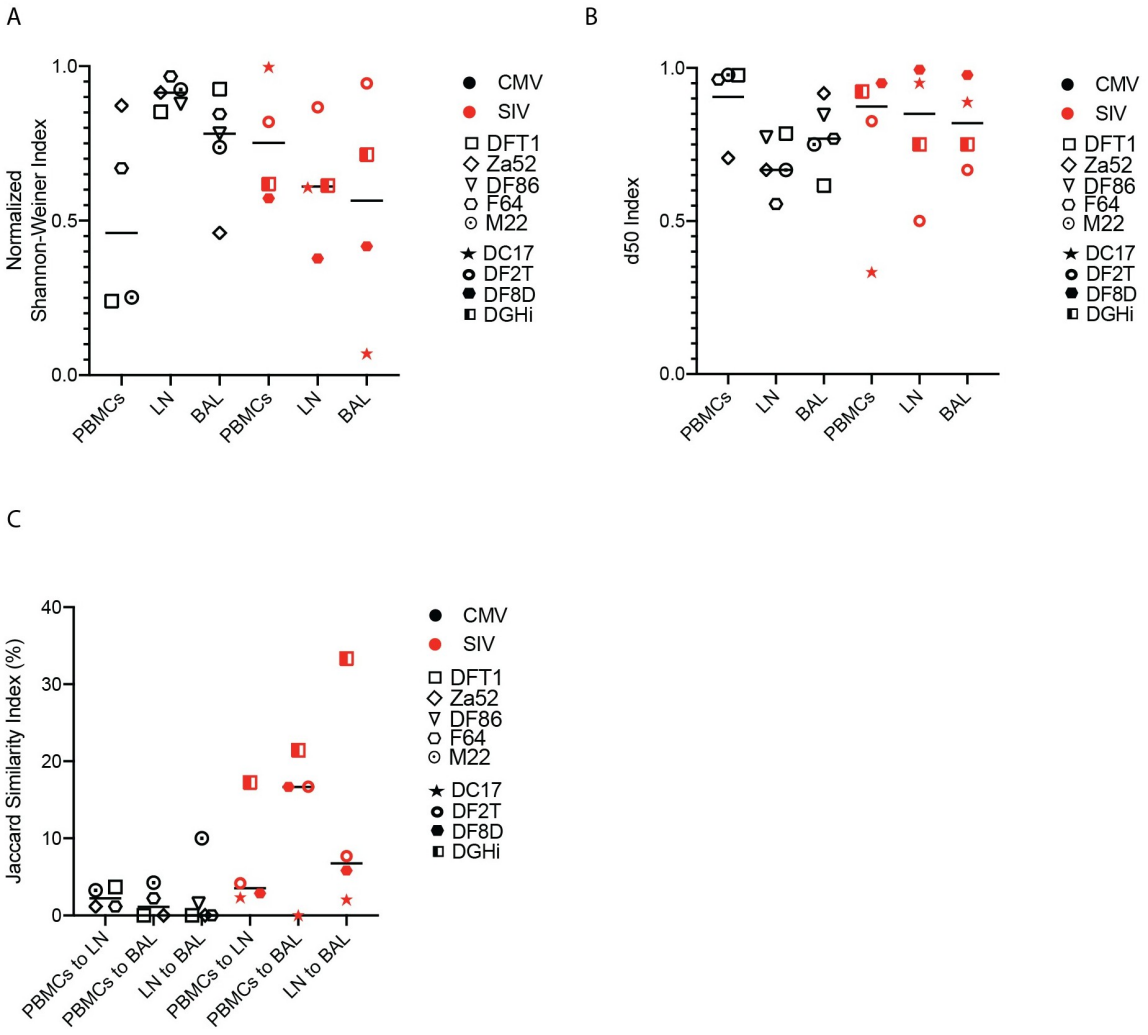
The TCR repertoires of SIV- and CMV- specific CD8^+^ T cells exhibit similar diversity and tissue similarity. PBMCs, LN and BAL were sampled from rhesus macaques who were chronically infected with SIVmac239X (without ARV treatment) or naturally infected with CMV. (A) The normalized Shannon-Weiner diversity index of the TCR repertoires. (B) The d50 diversity index of the TCR repertoires. (C) The Jaccard similarity index for comparisons between each tissue’s TCR repertoire. Data are presented as mean, with individual data points. One way ANOVA was used to determine statistical significance. n = 3 to 6 animals.

The Shannon diversity and d50 indexes of the clonotypes of chronically infected SIV- and CMV-specific CD8^+^ T cells were compared to evaluate virus-specific effects on their respective diversities. We found no significant differences in measures of diversity between these virus-specific CD8^+^ T cells in any anatomical site (Fig 5A and 5B). Jaccard similarity indexes of tissue comparisons, as a proxy measure of tissue specificity, were compared between CMV- and chronically infected SIV-specific CD8^+^ T cells and, similarly, showed no significant differences (Fig 5C). These comparisons suggest two pathogens that induce a chronic infection in the host can establish similarly diverse and tissue-specific clonotypic hierarchies.

### Virus-specific CD8^+^ T cells do not uniformly exhibit a tissue-resident phenotype

**Fig 6:**
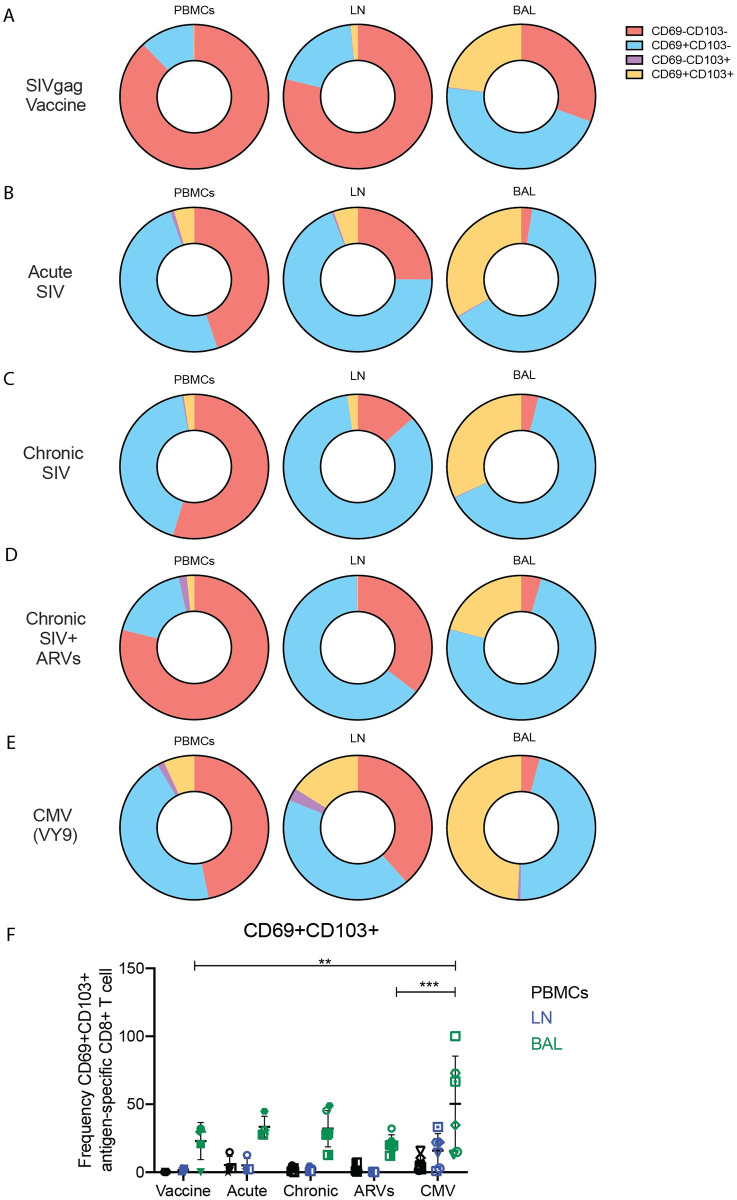
Expression patterns of CD69 and CD103 among virus-specific CD8^+^ T cells. The expression of CD69 and CD103 on antigen specific CD8^+^ T cells were assessed by flow cytometry across all experimental groups. (A-E) Average percentage of CD69^-^CD103^-^, CD69^+^CD103^-^, CD69^-^CD103^+^ and CD69^+^CD103^+^ antigen-specific (CM9) CD8^+^ T cells after exposure to SIV-gag DNA vaccine (A); during acute (B), chronic (C), and chronic SIVmac239X or SIVmac239 infection with ARV treatment (D); or during CMV infection (E). (F) The number of CD69^+^CD103^+^ antigen-specific CD8^+^ T cells during all experimental conditions. In (F) data are presented as mean with SD. Two-way ANOVA was used to determine statistical significance in (F). n = 3 to 6 animals.

Tissue-resident memory T cells (TRM) are frequently defined based upon expression patterns of CD69 and CD103 [25]. Presence of antigen in non-lymphoid tissues mediates the upregulation of CD69 and rapid formation of TRMs following multiple antigen stimulations [26, 27]. These cells are then retained in the tissues via release of local autocrine signals, such as transforming growth factor-β (TGFβ), where competition for these signals promotes diversity in TRM functionality [28,29]. In HIV infection, T cells expressing residency phenotypes dominate HIV-specific CD8^+^ T cells in lymphoid tissues and are present in high numbers in HIV elite controllers, suggesting they contribute to viral control [30]. Given the evidence of tissue-specific clonotypes of SIV- and CMV-specific CD8^+^ T cells, we sought to determine the proportion of antigen-specific CD8^+^ T cells that were expressing these TRM markers in PBMC, LNs and BAL of the animals we studied. Virus-specific CD8^+^ T cells expressing both CD69 and CD103 were infrequent in PBMC and LNs of all animals, irrespective of whether SIV- or CMV-specific CD8^+^ T cells were examined (Fig 6A–6E). Antigen-specific CD8^+^ T cells (either CMV- or SIV-specific) in the BAL were more frequently CD69^+^CD103^+^ (Fig 6A–6E), but VY9-specific CD8^+^ T cells in the BAL had a significantly higher percentage of CD69^+^CD103^+^ T cells compared to CM9-specific CD8^+^ T cells in after SIV gag DNA vaccination and chronic SIV infection with ARV treatment (Fig 6F). CD69 expression, without CD103 expression, was more frequent among antigen-specific CD8^+^ T cells in all anatomical sites (Fig 6).

## Discussion

Here we have studied the phenotypes and clonotypic hierarchies of CMV- and SIV-specific CD8^+^ T cells across multiple anatomical sites in rhesus macaques that were either virus-infected or vaccinated. In all cases we found evidence of tissue-specific CD8^+^ T cell clonotypes and the induction of public and shared TCR clonotypes. These data help explain the phenomenon of CD8^+^ T cell tissue-residency and demonstrate that either acute or chronic antigen exposure is required for tissue-resident CD8^+^ T cell maintenance *in vivo*.

Tissue-specific clonotypes have been identified in chronic SIV infection [9] and clonotypic discrepancies between the peripheral blood and other anatomical sites, such as lymphoid tissue, have been observed in chronic HIV infection [30]. During acute infection, clonal expansion of CD8^+^ T cells typically peaks at 4 weeks post infection [31], occurring during a period of reduced cytotoxicity, subsequent to initial increases in SIV-specific CD8^+^ T cells numbers [7]. The presence of public clonotypes in acute infection is suspected to reflect their large numbers in the naïve pool, due to the likely generation of these public clonotypes by convergent recombination in which the same amino acid can be encoded by multiple nucleotide sequences [32]. The presence of tissue-specific clonotypes during acute antigen exposure or acute infection suggest minimal trafficking between anatomical sites after priming. As these observations featured TCRs restricted to a singular viral epitope, confirmation with other SIV epitopes are required before confirming that all SIV-specific CD8^+^ T cells exhibit tissue specific clonotypes.

During chronic SIVmac239 infection, the TCR repertoires did not exhibit increased tissue similarity and showed significantly decreased diversity only in the BAL compared to acute infection. This differs from previous studies which showed that as SIV infection progresses to chronic infection, increased sharing of the TCR repertoire between tissues was observed along with decreased diversity and changes in clonal hierarchy in blood and mucosal tissues [33]. Furthermore, several studies have observed a narrowing of the TCR repertoire during ARV treatment in SIV and HIV infection, coinciding with reduced antigen presence [20,34]. We observed modest fluctuation of the repertoire throughout infection and with ARV treatment, with the emergence of novel clonotypes and changes in the dominant clones in multiple anatomical sites. Similar fluctuations have been observed previously among HIV-specific CD8^+^ T cells during ARV treatment [35]. These TCR fluctuations may be reflecting the response to emerging variant epitopes or low levels of viral reactivation during ARV treatment, or identification of existing clonotypes that were previously below the limit of detection, or recirculation from tissues we did not sample [36]. Given the emergence of tissue-specific novel clonotypes during chronic infection with ARV treatment, epitope variants may be disproportionally represented in different anatomical sites; therefore, analysis of viral sequences in particular tissues are merited.

We also assessed the CMV-specific CD8^+^ T cell repertoire. Similar to the clonotypic hierarchy of SIV-specific CD8^+^ T cells, CMV-specific CD8^+^ T cells also exhibited unique clonotypes in different anatomical sites; thus it is possible that any viral replication within individual tissues over prolonged periods may promote emergence of tissue epitope variants and differences in dominant epitope variants between tissues. Indeed, these virus-specific CD8^+^ T cells revealed similar diversity and tissue similarity, suggesting distinct viruses can induce similar clonotypic structures during chronic infection.

Given the evidence of tissue-specific clonotypes, we hypothesized that this may correlate with the presence of a TRM phenotype [25]. However, we did not find a preponderance of a TRM phenotype among the CD8^+^ T cells we studied. Indeed, the majority of antigen specific CD8^+^ T cells were not CD69^+^CD103^+^ regardless of anatomical site or antigen exposure. It is critical to note that CD103 is not expressed on all TRM [37] and use of another TRM marker such as CD49a [38] may have identified additional antigen-specific TRM.

Antigen-specific CD8^+^ T cells exhibit unique, tissue-specific clonotypes during acute infection, chronic infection and in presence of ARVs, with the TCR repertoire fluctuating throughout infection. Clonotypes unique to different anatomical sites are likely due to a general diversifying of the TCR repertoire during infection and are able to persist even when antigen load is reduced upon ARV treatment. The presence of tissue-specific clonotypes is not unique to SIV infection, with similar tissue-specificity identified in CMV-specific CD8^+^ T cells. Clonotypes unique to one anatomical site might suggest distinct CD8^+^ T cell phenotypes in each tissue, as a relationship between TCR sequence and phenotype has previously been observed using single cell RNA sequencing [39]. Moreover, unique cues provided within individual tissues may imprint individual phenotypic and functional attributes to antigen-specific CD8 T cells. These imply that surveying the antigen-specific CD8^+^ T cell repertoire by only sampling the blood may prevent identification of multiple TCR clonotypes only present in the tissues. Therefore, the data presented here suggests that sampling multiple anatomical sites is required to identify an accurate and comprehensive TCR repertoire in antigen-specific CD8^+^ T cells.

## Materials and methods

### Ethics statement

The National Institute of Allergy and Infectious Diseases (NIAID) animal care and use committee, as part of the National Institute of Health (NIH) intramural research program, approved all experimental procedures pertaining to the animals (protocol LVD 26E). The animals in this study were housed and cared for at the NIH animal center, under the supervision of the Association for the Assessment and Accreditation of Laboratory Animal Care (AAALAC)-accredited division of veterinary resources and as recommended by the office of animal care and use nonhuman primate management plan. Care at this facility met the standards set forth by the animal welfare act, animal welfare regulations, United States fish and wildlife services regulations, as well as the guide for the care and use of laboratory animals (8^th^ Edition). The physical conditions of the animals were monitored daily. Animals in this study were exempt from contact social housing due to scientific justification, per respective the NIAID/NIH institutional animal care and use committee (IACUC) protocol, and were housed in non-contact, social housing where primary enclosures consisted of stainless-steel primate caging. The animals were provided continuous access to water and offered commercial monkey biscuits twice daily as well as fresh produce, eggs and bread products and a foraging mix consisting of raisins, nuts and rice. Enrichment to stimulate foraging and play activity was provided in the form of food puzzles, toys, cage furniture, and mirrors.

### Study design

For the SIV infection model, nine uninfected Mamu-A*01+ rhesus macaques (*Macaca mulatta*) were intrarectally challenged with SIVmac239X or infected i.v with SIVmac239. Once viral load was detected in the plasma as described [40], animals were sedated with Telazol at 3–4 mg/kg i.m. and peripheral blood, BAL and biopsies of LNs were taken from animals in acute infection (approximately 10 days to three months after infection), chronic infection (several months after infection) and between two and seven months after administration of combination ARVs (S1 Table) as previously described [40]. ARV treatment consisted of a previously described regimen [41], including the s.c administration of nucleo(s/t)ide reverse transcriptase inhibitors emtricitabine (FTC) and tenofovir disoproxil fumarate [TDF, prodrug of tenofovir (TFV, PMPA)] with the integrase strand transfer inhibitor dolutegravir (DTG). For the vaccination model, five SIV-uninfected Mamu-A*01^*+*^ rhesus macaques (S2 Table) were administered, intramuscularly via Pharmajet (Golden, CO, USA), 1 mg of a vaccine consisting of a DNA plasmid containing the SIV gag gene, driven by the CMV promoter [16]. The SIV-gag vaccine was administered 5 times to each animal at days 0, 28, 56, 84, and approximately day 211 post first dose. PBMCs, BAL and biopsies of LNs and jejunum were taken from animals before and 10 days after final DNA vaccination. For CMV-specific CD8^+^ T cells, Mamu-A*02^*+*^ rhesus macaques (S3 Table) were identified as being CMV-infected via serology. PBMCs, BAL and biopsies of LNs and liver were obtained to study the resident CMV-specific CD8^+^ T cells. Single-cell suspensions were generated from all blood draws and biopsied tissues and were used for flow cytometric analysis and sorting. Animal details are included in S1–S3 Tables.

### Flow cytometry and sorting SIV-specific CD8^+^ T cells

Single-cell suspensions were washed twice with PBS (PBMCs) or RPMI 1640 medium supplemented with 10% fetal bovine serum, 2 mM l-glutamine, and 1% penicillin/streptomycin (R10 media) (all from HyClone, GE Healthcare Life Sciences). SIV-specific CD8^+^ T cells were identified by CM9 (CTPYDINQM; residues 181–189 of SIV Gag protein) conjugated MHC Class I Pentamers (Proimmune). CMV-specific CD8^+^ T cells were identified by AN10 (TTRSLEYKN, residues 279–288 of CMV IE2 protein) and VY9 (VTTLGMALY, residues 134–142 of CMV IE1 protein) MHC-I Pentamers (NIH tetramer facility and ProImmune respectively) [24]. All tetramers were conjugated to the APC fluorophore. Antibodies against cell surface markers utilized to identify CD8^+^ T cells are included in S4 Table. Dead cells were excluded using Live/dead Aqua dead cell stain kit (ThermoFisher). SIV-specific or CMV-specific CD8^+^ T cells were sorted using an S6 Symphony Cell Sorter (BD). CD8^+^ T cell phenotypes were also assessed via flow cytometry using a Fortessa cytometer (BD). Primary gating strategy is displayed in S4 Fig. Flow cytometry data was analyzed in FlowJo 10.8.1.

### Clonotype analysis

Between 30 and 10,000 epitope-specific CD8^+^ T cells were sorted into 100 μL of RNAlater (MilliporeSigma). Given the oligoclonal nature of the epitope-specific CD8 T cell clonotypic hierarchy, even analysis of approximately 100 cells has been shown to capture the numbers of individual clonotypes [30,42–44]. TCR CDR3 regions were amplified without bias using template-switch anchored reverse transcription PCR, as described previously [45]. Unique barcodes and the P5 and P7 Illumina sequencing adaptors (Illumina) were added to all PCR products with sequential PCRs. Any samples that failed quality control measures after barcoding were eliminated. Sequences were generated by next-generation sequencing (Illumina) as previously described [46]. Clonotypes were aligned and TCRΒV and TCRΒJ segments were identified using MiXCR software (MiLaboratory). All diversity and similarity indices were determined with VDJTools (Mikhail Shugay). VDJTools was also utilized for graphing of the V and J segment usage. Graphing of MDS plots was conducted in R Studio v1.3.1056 with raw data generated from VDJTools. We defined clonotypes by the V and J segment [47] in addition to the CDR3 amino acid sequence. We define “public” clonotypes as the same clonotype (same V and J segments and same CDR3 amino acid sequence) found in multiple animals in this study, and matching clonotypes previously identified in the VDJdb database (https://vdjdb.cdr3.net). “Shared” clonotypes were those found only in one animal but observed in multiple tissues. “Private” clonotypes were identified in a single anatomical site in a single animal. TCR repertoires plots represent all clonotypes that constitute more than 1% of the TCR repertoire. In instances were only one tissue/animal/timepoint could be obtained and those samples had insufficient cell numbers or failed quality control measures, the samples were eliminated from analysis and are absent from the corresponding graphs.

### Statistics

All statistical analyses were conducted in Graphpad Prism v8. Statistical analyses of multiple experimental groups were conducted by one or two-way ANOVAs or mixed effects analysis as appropriate. Multiple comparisons tests were conducted with each ANOVA or mixed effects analysis. Significance is defined as p < 0.05. p values of less that 0.05 are denoted as * and p values of less than 0.01 are denoted as **.

## Supporting information

S1 Fig The number of SIV-specific CD8^+^ T cells and V and J segment usage is consistent throughout SIV infection. PBMCs, LN biopsies and BAL were sampled from SIVmac239X or SIVmac239-infected rhesus macaques during acute infection, chronic infection and after 2–7 months of ARV treatment. (A) The number of SIV-specific CD8^+^ T cells in multiple anatomical sites at all time points, as a percentage of total CD8^+^ cells. Data are presented as mean with SD and individual data points. (B) Heatmap of the V segments of the TCRB genes in multiple anatomical sites at all time points. (C) Heatmap of the J segments of the TCRB genes in multiple anatomical sites at all time points. Mixed effects analysis was used to determine statistical significance in (A). n = 7–9 animals.
(TIF)

S2 Fig The V and J segment usage in TCR repertoires of vaccine-induced SIV-specific CD8^+^ T cells across multiple anatomical sites. PBMCs, LN and BAL were sampled from Rhesus macaques who had been administered with SIV-gag DNA vaccine. (A) Heatmap of the V segments of the TCRB genes in multiple anatomical sites. (B) Heatmap of the J segments of the TCRB genes in multiple anatomical sites. (C) MDS plot of the TCR repertoires of SIV-specific CD8^+^ T cells from multiple anatomical sites. n = 4 animals.
(TIF)

S3 Fig The V and J segment usage in TCR repertoires of VY9-specific CD8^+^ T cells in multiple anatomical sites. PBMCs, LN, liver biopsies, and BAL were sampled from Rhesus macaques who had been naturally infected with CMV. (A) Heatmap of the V segments of the TCRB genes in multiple anatomical sites. (B) Heatmap of the J segments of the TCRB genes in multiple anatomical sites. n = 5 animals.
(TIF)

S4 Fig Gating strategy for identifying antigen-specific CD8^+^ T cells. (A) Identification of CM9 tetramer positive cells via identification of lymphocytes, singlets, live cells, and CD8^+^NKG2a^-^ cells. Gating sequence is indicated by black arrows. (B) Gating strategy for identification of CD69^-^CD103^-^, CD69^+^CD103^-^, CD69^-^CD103^+^ and CD69^+^CD103^+^ antigen-specific (CM9) CD8^+^ T cells after identification of CM9^+^ CD8^+^ T cells (as described in (A)). Gating sequence is indicated by black arrows.
(TIF)

S1 Table Animal details—SIV infection study. Details of animals included in the SIV infection study. “~” denotes viral loads measured 2–3 days before/after the timepoint sampled. “*” denotes animals that were also used for the vaccination study several months prior to infection.
(DOCX)

S2 Table Animal details—SIV vaccination study. Details of animals included in the SIV vaccination study.
(DOCX)

S3 Table Animal details—CMV infection study. Details of animals included in the CMV infection study.
(DOCX)

S4 Table Flow cytometry antibodies. List of antibodies used in flow cytometric analysis and sorting.
(DOCX)
