# Supplementary figures and images for: Multiple modes of antigen exposure induce clonotypically diverse epitope-specific CD8+ T cells across multiple tissues in nonhuman primates
Source: PLoS Pathog. 2022 Jul 7;18(7):e1010611. doi: 10.1371/journal.ppat.1010611 (PMC9262242; doi:10.1371/journal.ppat.1010611)

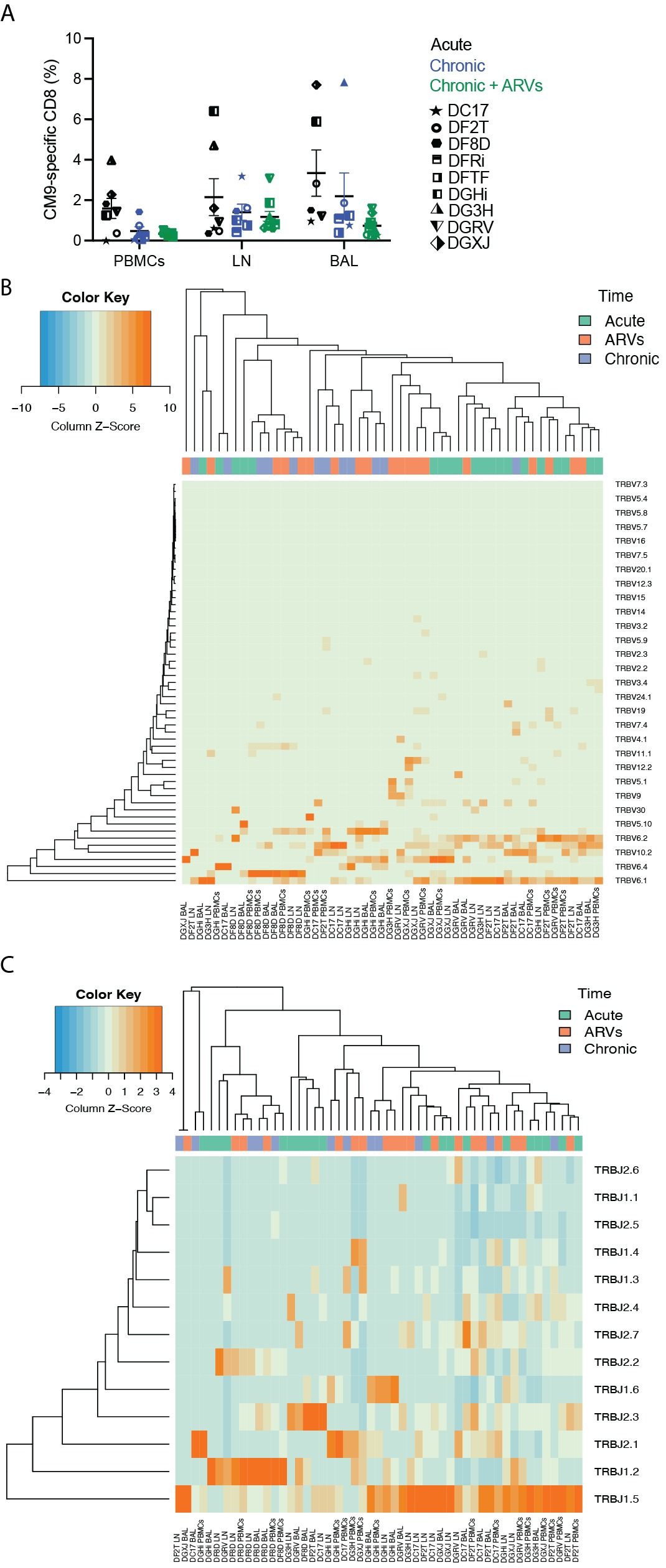

Supplement: S1 Fig — PBMCs, LN biopsies and BAL were sampled from SIVmac239X or SIVmac239-infected rhesus macaques during acute infection, chronic infection and after 2–7 months of ARV treatment. (A) The number of SIV-specific CD8+ T cells in multiple anatomical sites at all time points, as a percentage of total CD8+ cells. Data are presented as mean with SD and individual data points. (B) Heatmap of the V segments of the TCRB genes in multiple anatomical sites at all time points. (C) Heatmap of the J segments of the TCRB genes in multiple anatomical sites at all time points. Mixed effects analysis was used to determine statistical significance in (A). n = 7–9 animals. (TIF) [file ppat.1010611.s001.tif]

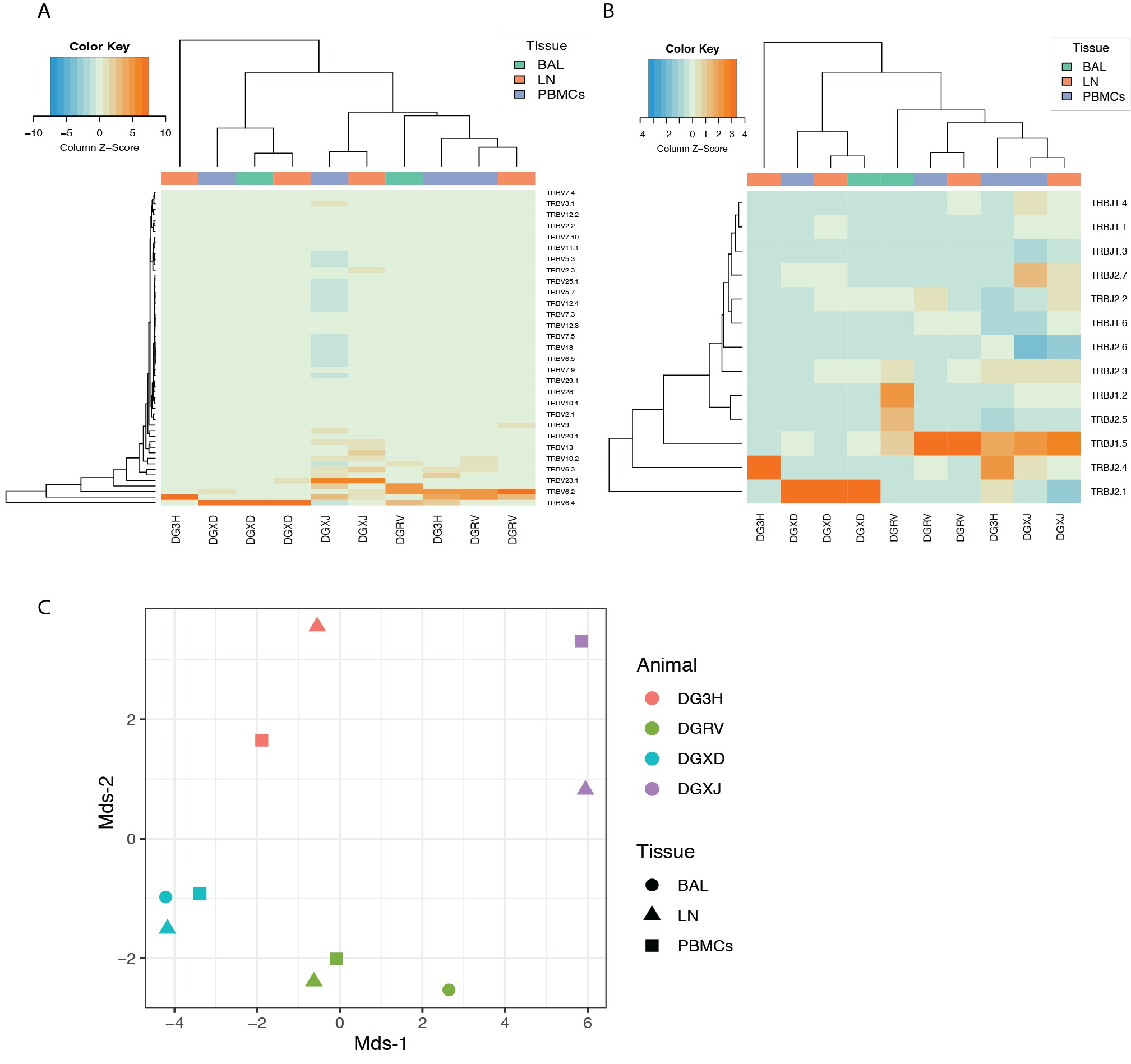

Supplement: S2 Fig — PBMCs, LN and BAL were sampled from Rhesus macaques who had been administered with SIV-gag DNA vaccine. (A) Heatmap of the V segments of the TCRB genes in multiple anatomical sites. (B) Heatmap of the J segments of the TCRB genes in multiple anatomical sites. (C) MDS plot of the TCR repertoires of SIV-specific CD8+ T cells from multiple anatomical sites. n = 4 animals. (TIF) [file ppat.1010611.s002.tif]

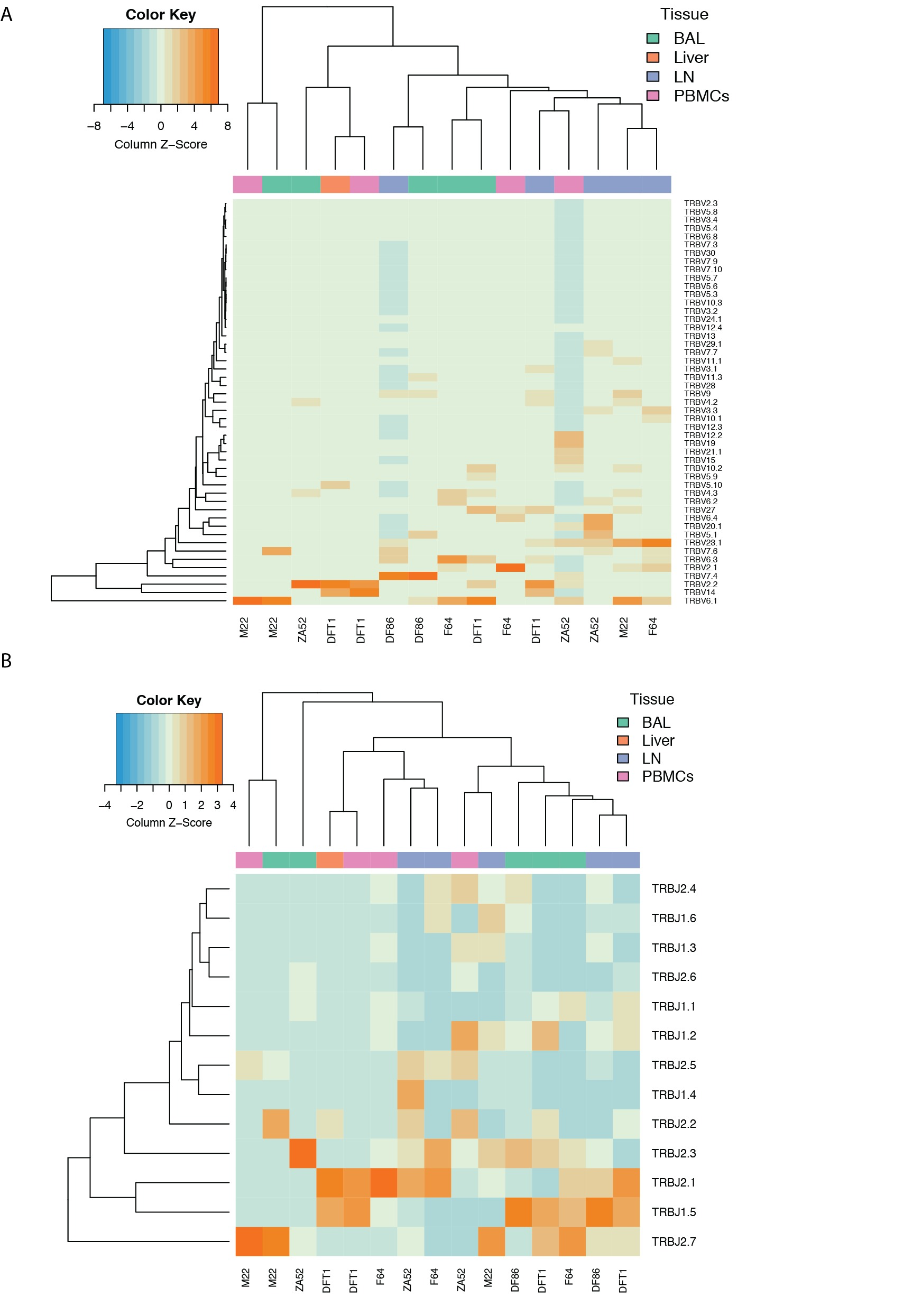

Supplement: S3 Fig — PBMCs, LN, liver biopsies, and BAL were sampled from Rhesus macaques who had been naturally infected with CMV. (A) Heatmap of the V segments of the TCRB genes in multiple anatomical sites. (B) Heatmap of the J segments of the TCRB genes in multiple anatomical sites. n = 5 animals. (TIF) [file ppat.1010611.s003.tif]

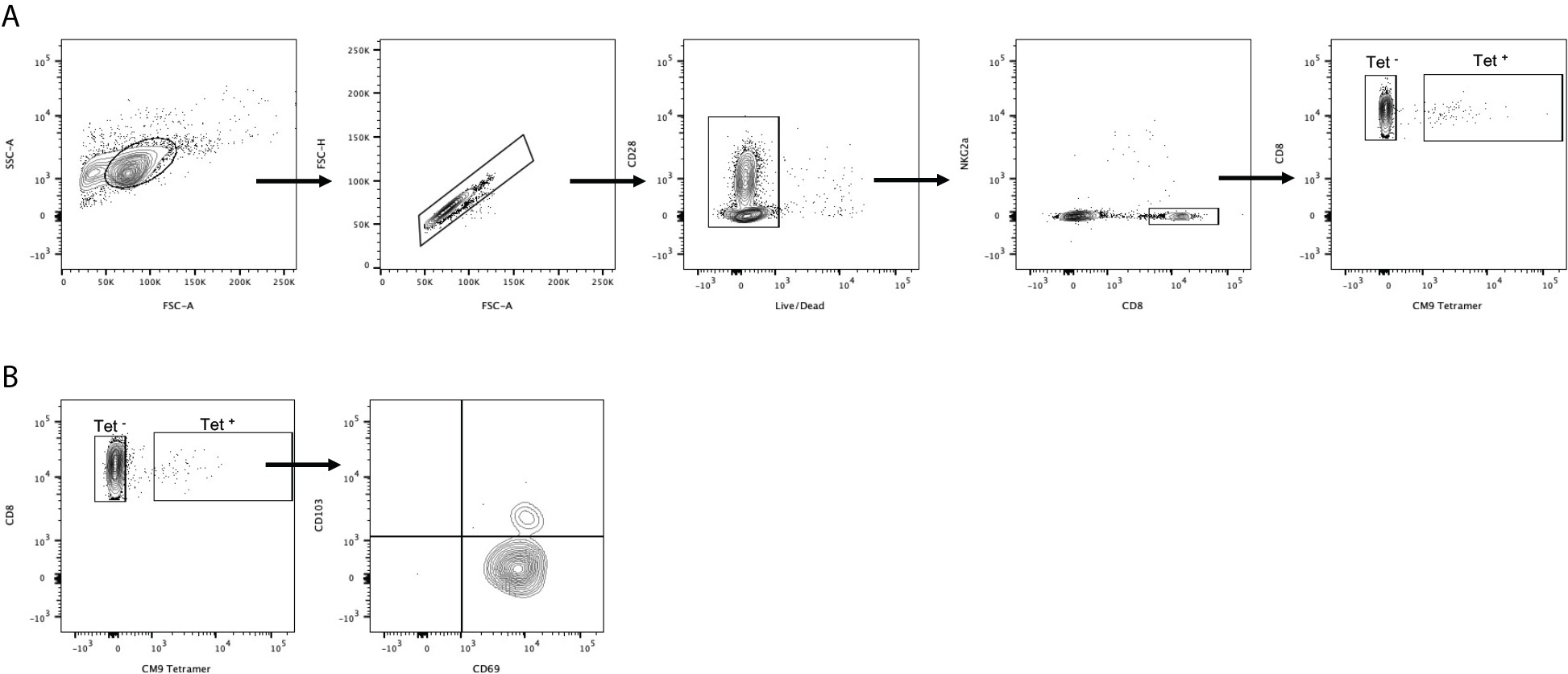

Supplement: S4 Fig — (A) Identification of CM9 tetramer positive cells via identification of lymphocytes, singlets, live cells, and CD8+NKG2a- cells. Gating sequence is indicated by black arrows. (B) Gating strategy for identification of CD69-CD103-, CD69+CD103-, CD69-CD103+ and CD69+CD103+ antigen-specific (CM9) CD8+ T cells after identification of CM9+ CD8+ T cells (as described in (A)). Gating sequence is indicated by black arrows. (TIF) [file ppat.1010611.s004.tif]
