# Supplementary material for: Multiple modes of antigen exposure induce clonotypically diverse epitope-specific CD8+ T cells across multiple tissues in nonhuman primates
Source: PLoS Pathog. 2022 Jul 7;18(7):e1010611. doi: 10.1371/journal.ppat.1010611 (PMC9262242; doi:10.1371/journal.ppat.1010611)
Supplement: S1 Table — Details of animals included in the SIV infection study. “~” denotes viral loads measured 2–3 days before/after the timepoint sampled. “*” denotes animals that were also used for the vaccination study several months prior to infection. (DOCX) [file ppat.1010611.s005.docx]

**Supplementary Table 1: Animal details – SIV infection study**

| Animal | MHC  Allele | Virus | Time  point | Days post infection | Viral load  SIVg copies/ml | Lymphocyte count (/ml) | Symbol in Figures |
| --- | --- | --- | --- | --- | --- | --- | --- |
| DC17 | MamuA*01 | SIVmac239X | Acute | 65 | 2,000,000 | 3339 |  |
| DC17 | MamuA*01 | SIVmac239X | Chronic | 267 | 2,600,000 | 3492 |  |
| DC17 | MamuA*01 | SIVmac239X | Chronic+ARVs | 372 | 4200 | 6572 |  |
| DF2T | MamuA*01 | SIVmac239X | Acute | 32 | ~ 150,000 | 2652 |  |
| DF2T | MamuA*01 | SIVmac239X | Chronic | 203 | 11,000,000 | 1452 |  |
| DF2T | MamuA*01 | SIVmac239X | Chronic+ARVs | 308 | 65 | 2378 |  |
| DF8D | MamuA*01 | SIVmac239X | Acute | 56 | 130,000 | 2184 |  |
| DF8D | MamuA*01 | SIVmac239X | Chronic | 237 | 1,500,000 | 1330 |  |
| DF8D | MamuA*01 | SIVmac239X | Chronic+ARVs | 348 | 40 | 2444 |  |
| DGHi | MamuA*01 | SIVmac239X | Acute | 51 | ~ 58,000 | 4590 |  |
| DGHi | MamuA*01 | SIVmac239X | Chronic | 245 | 500 | 3813 |  |
| DGHi | MamuA*01 | SIVmac239X | Chronic+ARVs | 364 | Below LOD | 3200 |  |
| DFRi | MamuA*01 | SIVmac239X | Chronic | 124 | 950,000 | 1482 |  |
| DFRi | MamuA*01 | SIVmac239X | Chronic+ARVs | 237 | Below LOD | 1650 |  |
| DFTF | MamuA*01 | SIVmac239X | Chronic | 292 | 8,000,000 | 1638 |  |
| DFTF | MamuA*01 | SIVmac239X | Chronic+ARVs | 397 | 320 | 2688 |  |
| DG3H* | MamuA*01 | SIVmac239 | Acute | 20 | 82000 | 1932 |  |
| DG3H* | MamuA*01 | SIVmac239 | Chronic+ARVs | 241 | Below LOD | 2668 |  |
| DGRV* | MamuA*01 | SIVmac239 | Acute | 10 | 23000 | 1677 |  |
| DGRV* | MamuA*01 | SIVmac239 | Chronic+ARVs | 227 | Below LOD | 3468 |  |
| DGXJ* | MamuA*01 | SIVmac239 | Acute | 20 | 2300000 | 3738 |  |
| DGXJ* | MamuA*01 | SIVmac239 | Chronic+ARVs | 227 | 65 | 5586 |  |
