# Supplementary material for: Multiple modes of antigen exposure induce clonotypically diverse epitope-specific CD8+ T cells across multiple tissues in nonhuman primates
Source: PLoS Pathog. 2022 Jul 7;18(7):e1010611. doi: 10.1371/journal.ppat.1010611 (PMC9262242; doi:10.1371/journal.ppat.1010611)
Supplement: S2 Table — Details of animals included in the SIV vaccination study. (DOCX) [file ppat.1010611.s006.docx]

**Supplementary Table 2: Animal details – SIV vaccination study**

| Animal | MHC Allele | Vaccine | Time point | Lymphocyte count (/ml) | Symbol in Figures |
| --- | --- | --- | --- | --- | --- |
| DG3H | MamuA*01 | DNA plasmid/R-SIVgag vaccine | 10 days post last dose | 2576 |  |
| DGXD | MamuA*01 | DNA plasmid/R-SIVgag vaccine | 10 days post last dose | 4620 |  |
| DGRV | MamuA*01 | DNA plasmid/R-SIVgag vaccine | 10 days post last dose | 3850 |  |
| DGPZ | MamuA*01 | DNA plasmid/R-SIVgag vaccine | 10 days post last dose | 4200 |  |
| DGXJ | MamuA*01 | DNA plasmid/R-SIVgag vaccine | 10 days post last dose | 4200 |  |
