# Supplementary material for: Multiple modes of antigen exposure induce clonotypically diverse epitope-specific CD8+ T cells across multiple tissues in nonhuman primates
Source: PLoS Pathog. 2022 Jul 7;18(7):e1010611. doi: 10.1371/journal.ppat.1010611 (PMC9262242; doi:10.1371/journal.ppat.1010611)
Supplement: S3 Table — Details of animals included in the CMV infection study. (DOCX) [file ppat.1010611.s007.docx]

**Supplementary Table 3: Animal details – CMV infection study**

| Animal | MHC Allele | CMV Infection | Reactive CMV epitope | Lymphocyte count (/ml) at day of sampling | Symbol in Figures |
| --- | --- | --- | --- | --- | --- |
| DGXR | MamuA*02 | Positive | AN10 | 2184 |  |
| DFT1 | MamuA*02 | Positive | AN10 | 1452 |  |
| Za52 | MamuA*02 | Positive | AN10 | 2592 |  |
| DGZi | MamuA*02 | Positive | AN10 | 3763 |  |
| DFT1 | MamuA*02 | Positive | VY9 | 1892 |  |
| DF86 | MamuA*02 | Positive | VY9 | 1643 |  |
| Za52 | MamuA*02 | Positive | VY9 | 2592 |  |
| F64 | MamuA*02 | Positive | VY9 | 1911 |  |
| M22 | MamuA*02 | Positive | VY9 | 2679 |  |
| H788 | MamuA*02 | Positive | VY9 | 552 |  |
