# Supplementary material for: Multiple modes of antigen exposure induce clonotypically diverse epitope-specific CD8+ T cells across multiple tissues in nonhuman primates
Source: PLoS Pathog. 2022 Jul 7;18(7):e1010611. doi: 10.1371/journal.ppat.1010611 (PMC9262242; doi:10.1371/journal.ppat.1010611)
Supplement: S4 Table — List of antibodies used in flow cytometric analysis and sorting. (DOCX) [file ppat.1010611.s008.docx]

**Supplementary Table 4: Flow Cytometry Antibodies.**

| Antigen | Fluorochrome | Clone | Supplier | Catalog Number |
| --- | --- | --- | --- | --- |
| CD103 | FITC | B-Ly7 | ThermoFisher | 11-1038-42 |
| CD8 | Pacific Blue | RPA-T8 | BD | 558207 |
| NKG2a (CD159a) | PE | Z199 | Beckman Coulter | 1M3291U |
| CD28 | ECD | CD28.2 | Beckman Coulter | 6607111 |
| CD69 | PE Cy7 | FN50 | BD | 5577454 |
| CD3 | PerCp5.5 | Sp342 | BD | 552852 |
| CD4 | BV650 | OKT4 | Biolegend | 317436 |
| CD45 | AL700 | D058-1283 | BD | 561288 |
| CD95 | PECy5 | DX2 | Biolegend | 305610 |
| CD20 | APCH7 | 2H7 | BD | 560734 |
| CD49a | PE (not used in same panel as NKG2a) | TS2/7 | Biolegend | 328303 |
| Live/dead stain | Aqua Blue | - | Invitrogen | L34957 |
